# Epidemiological profile of visceral leishmaniasis in northern Morocco (2009-2018)

**DOI:** 10.11604/pamj.2024.48.87.41141

**Published:** 2024-07-04

**Authors:** Kaoutar Nabah, Nadya Mezzoug, Halima Oufdou, Kacem Rharrabe

**Affiliations:** 1 Laboratory of Biology, Environment and Sustainable Development, Higher Normal School, Abdelmalek Essaadi University, Tetouan, Morocco,; 2 Laboratory of Chemistry Applied Microbiology and Biotechnology, Faculty of Sciences, Abdelmalek Essaadi University, Tetouan, Morocco,; 3 Laboratory of Applied Economics, Faculty of Economic and Social Legal Sciences, Mohammed V University, Rabat, Morocco

**Keywords:** Epidemiology, neglected tropical disease, visceral leishmaniasis, Morocco

## Abstract

**Introduction:**

visceral leishmaniasis (VL) represents the most serious and severe form of leishmaniasis in Northern Morocco. In this context, the objective of this study was to describe the epidemiological profile of VL in the Tangier Tetouan Al-Hoceima region from 2009 to 2018.

**Methods:**

the epidemiologic data was collected from April 28^th^, 2019 to February 2^nd^, 2020 from files and investigation reports of cases. Additionally, annual reports for VL from the health services and provincial laboratories of parasitology were consulted. The analysis was conducted using statistical package for the social sciences (SPSS) v26 software.

**Results:**

the study included 304 cases. Chefchaouen province was the highest endemic area (54.5%). The cases in the spring reached 36.5% and were characterized by age ≤5 years old (78.8%), male gender (M/F=1.3) and rural residents (91.4%). The number of inhabitants per household of cases was >5 persons (68.5%). A total of 94.3% and 98% had no suspect cases around or in their homes, respectively. Farmers accounted for 74.5% of cases. Signs of fever were present in 17.4% of cases, with 67.3% of these cases presenting these signs for a duration of more than 30 days. A total of 64.2% cases were diagnosed within a month. The serological test was used for diagnosis in 67.1% of cases and for the treatment, glunantime® was used in all cases (100%).

**Conclusion:**

to eliminate the VL infection, it's necessary to monitor the entomological, mammalogical investigation. Also, to activate the Integrated Vector Control Management Committee at the most endemic province and to inform the community as well as the professionals of health about the VL control measures. A correlational study of the VL socio-economic and climate factors is recommended.

## Introduction

Visceral leishmaniasis (VL) is a neglected tropical disease (NTD) that can become fatal in the absence of adequate treatment. The World Health Organization, has set the goal of eliminating 85% of VL infections in endemic countries by the year 2030 [1]. Annually, VL infection affects 50,000 to 90,000 new cases worldwide, with only 24-45% of cases being reported [2]. In 2019, 90% of cases occurred in Ethiopia, Eritrea, India, Iraq, Kenya, Nepal, Somalia, South Sudan, and Sudan [3]. The vital VL infection due to Leishmania parasite (*L. infantum, L. chagasi, L. donovani*) and much more by other species of cutaneous leishmaniasis such as *L. tropica* [4-6]. In general, two noso-geographical types of VL are detected: the anthroponotic (kala-azar) and the zoonotic VL [7]. The first is due to *L. Donovani* and spreads in Eastern Africa and the Indian subcontinent [7]. The second is caused by *L. infantum*, and it has appeared in the Middle East, South America and the Mediterranean region [7]. In these last areas, 600 to 2000 VL cases are registered per year [8]. Although, 85% of VL cases are declared only in Iraq [8]. In Morocco, VL has been resurgence since the 1990s, and it is a notifiable disease since 1995 according to the Ministerial letter (No. 68395) of the Health and Social Protection Ministry (MHSP) [9]. Two years later, in 1997, the health program for leishmaniasis control was launched [9]. For its distribution, the VL infection is rife in subhumid areas of the north and is spreading towards the center of Morocco [10-12]. It´s known, caused by *L. infantum* as a zoonotic infection in the country [12-14]. In 2014, the national incidence was at 0.4 cases per 100000 habitants [12]. Yearly, the VL number of cases is estimated at an average of 100 cases per year [10-12]. From 2008 to 2017, the VL constituted 3% of leishmaniasis cases [15]. In 2019, its reported 91 cases of VL and in 2020, only 69 cases have been declared with an underestimate of 50% of cases due to COVID-19 outbreak [3,16]. In the same year (2020), 14 cases were reported in the Tangier Tetouan Al-Hoceima region (TTA) in northern Morocco [16]. This region includes the most active VL focus in Morocco [12,17]. In fact, Chefchaouen province of TTA region has been maintaining the first endemic position at regional and national levels [12,17]. Up to now, the VL Morocco program is a passively reporting cases [9]. Diagnosis confirmation is based on serology examination and the treatment of the pentavalent antimonial group [9,18]. The aim of our study is to describe the epidemiological profile of VL infection in the most affected region (TTA) of the country from 2009 to 2018 by studying the new characteristics of the infection.

## Methods

**Study design:** our epidemiological study is a descriptive retrospective and transversal study employing a quantitative approach from 2009 to 2018.

**Setting:** our epidemiological study is carried out from 28/04/2019 to 18/02/2020 in the TTA region. This region had a surface area of 16 010 km^2^ with 3 857 442 inhabitants in 2021 and includes 8 provinces (Ouezzane, Tetouan, Tangier-Assilah, Larache, Fahse-Anjra, M´Diq- Fnideq, Chefchaouen and Al-Hoceima) [13]. It contributes to 25.2% of the Gross Domestic Product [19].

**Participants:** all declared positive cases of VL infection to the Ministry of Health and social protection by Tangier Tetouan Al-Hoceima regional direction.

**Variables:** the examined variables were qualitative and quantitative. It were related to different aspects. The spatial-temporal aspect was considered in the distribution by province, months, years, seasons, and incidence of cases. The sociodemographic and economic aspects were described in terms of age, areas of provenance, gender, number of inhabitants per household, duration of habitation, secondary residence, movement out of locality profession, and type of profession. The entourage and clinical aspects were measured by the known cases in home, known cases in surrounding, suspects cases in home, dogs at home, dog in locality, rodents in homes or entourage, diagnostic test, delay of diagnosis, clinical signs, fever duration and treatment variables.

**Data collection:** data for the years 2009-2018 were collected from the files and survey reports around the cases of VL. The complete data were extracted from provincial parasitology laboratory reports, as well as from termly and annual reports. The Primary Health Care Network of the Provincial Health Delegation and the Parasitic Diseases Department of the Ministry of Health and Social Protection (MHSP) were constituted the establishments where the data were obtained. All the Data was included in Excel software.

**Study size:** all the 304 declared VL cases in the TTA region during the period of study were included.

**Statistical methods:** a descriptive data analysis was conducted before for all the collected epidemiology Data. SPSS v.24 (IBM SPSS Advanced Statistics 24.0 Z125-5543-05), XLstat and Excel were used to do the analysis.

## Results

**Figure 1:**
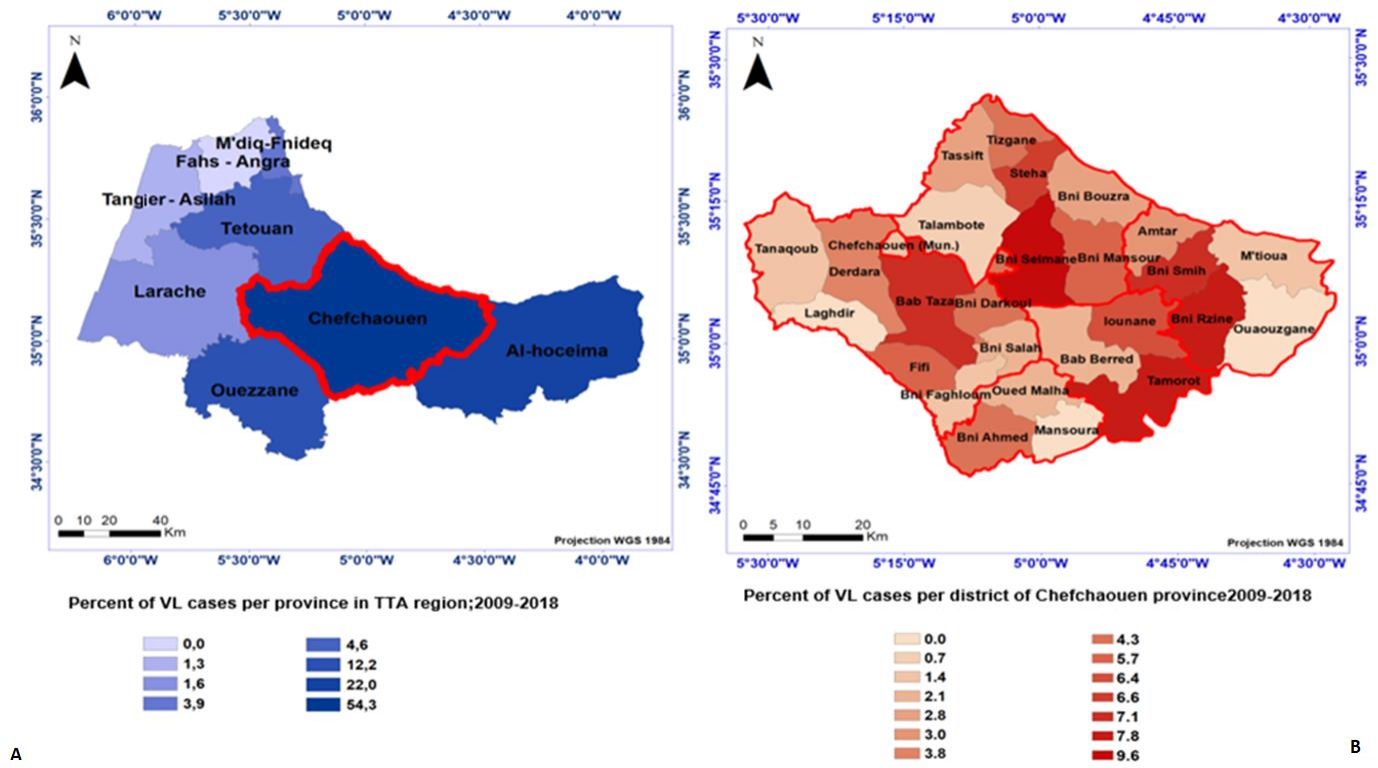
spatial distribution of visceral leishmaniasis cases in Tangier Tetouan Al-Hoceima region by province; A) by Chefchaouen district; B) 2009-2018

**Spatial distribution:** in the TTA region, 304 cases were included in the study during 2009-2018 (Table 1). A total of 98.7% of the cases were passively detected and 85% of the cases were indigenous origin. A total of 165 cases (54.3%) were recorded in the province of Chefchaouen, 67 of cases in province of Al-Hoceima (22%) and 37 of cases in Ouezzane (12.2%) (Table 1, Figure 1A). At Chefchaouen, the district of Bni Selmane is the most affected and has recorded 9.6% of the province cases (Figure 1B).

**Table 1:** spatial-temporal characteristics of VL cases in TTA region; 2009-2018

Variable(n)	Frequency	Percent (%)
**Screening type (n=304)**		
Active	4	1.4
Passive	299	98.7
**Origin of infection (n=287)**		
Indigenous	244	85.0
Imported	43	15,0
**Province (n=304)**		
Al-Hoceima	67	22.0
Chefchaouen	165	54.3
Larache	5	1.6
M'diq-Fnideq	12	3.9
Ouezzane	37	12.2
Fahse-Anjra	0	0.0
Tangier-Assilah	4	1.3
Tetouan	14	4.6
**Years (n=304)**		
2009	33	10.9
2010	47	15.5
2011	40	1.2
2012	34	11.2
2013	37	12.2
2014	18	5.9
2015	18	5.9
2016	33	10.9
2017	26	8.6
2018	18	5.9
**Months (n=304)**		
January	23	7.6
February	37	12.2
March	44	14.5
April	35	11.5
May	32	10.5
June	25	8.2
July	29	9.5
August	15	4.9
September	14	4.6
October	18	5.9
November	17	5.9
December	15	4.9
**Seasons (n=304**)		
Autumn	49	16.1
Summer	69	22.7
Winter	75	24.7
Spring	111	36.5

**Figure 2:**
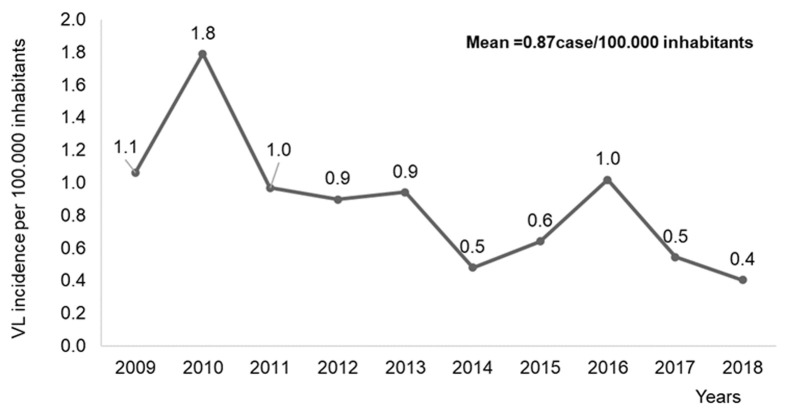
distribution of visceral leishmaniasis incidence in Tangier Tetouan Al-Hoceima region; 2009-2018

**Temporal distribution:** in 2010, the maximum number of cases in the region was recorded. This figure was 47 cases, which represents 15.5% of the total cases in the period 2009-2018 (Table 1). The highest incidence of the disease in the TTA region was observed in 2010, with 1.8 cases per 100,000 inhabitants (Figure 2) and the annual mean of VL incidence was 0.87 cases per 100,000 inhabitants (Figure 2). The monthly distribution of cases showed that 14.5% of cases (44) were recorded in March (Table 1). The season with the highest number of cases was spring, with 111 cases, followed by winter, with 75 cases, representing 36.5% and 24.7% of the total, respectively (Table 1).

**Socio-economic characteristics:** Table 2 shows that 78.8% of VL cases (235) were ≤ 5 years old. The median age was 2 (1, 4) years. The sex ratio (M/F=1.3) was in favor of the Males and a total of 277 of cases were in rural areas, which represents 91.4% of total cases (Table 2). The number of inhabitants per household was >5 persons for 68.5% of the total cases. Cases without profession accounted for 66. 5% of the cases (109), while the cases with profession were farmers in the majority (74.5% of 41 cases) (Table 2).

**Table 2:** socio-economic and clinical characteristics of VL cases in TTA region; 2009-2018

Variable(n)	Frequency	Percent(%)
**Age(n=298)**		
≤5years	235	78.8
>5years	63	21.2
**Gender (n=304)**		
Female	131	43.1
Male	173	56.9
**Residence (n=303)**		
Rural	277	91.4
Urban	26	8.5
**Profession(n=64)**		
Without	109	66.5
With	55	33.5
**With profession(n=41)**		
Farmers	41	74.5
Students	5	9.1
Workers	9	16.4
**Duration of residence (n=111)**		
≤1 year	27	24.3
>1year	84	75.7
**Number of persons per household (n=165)**		
≤5 persons	52	31.5
>5 persons	113	68.5
**Secondary residence (n=186)**		
No	180	96.8
Yes	6	3.2
**Movement out of locality (n=191)**		
No	172	90.1
Yes	19	9.9
**Affected for the first time (n=212)**		
No	2	0.9
Yes	210	99.1
**Clinical signs (195)**		
Fever	191	17.4
Pallor	190	17.3
Splenomegaly	190	17.3
Adenopathy	118	10.7
Slimming	173	15.7
Hepatomegaly	129	11.7
Anemia	109	9.9

**Demographic characteristics:** the median duration of residency in the localities of cases was 2 (1-3) years. The percent of 96.8% of cases (180) had no secondary residence outside their localities. Added that, 172 of cases were not traveling at the time of the infection onset (Table 2).

**Clinical characteristics:** the majority (99.1%) of the cases (210) were affected for the first time (Table 2). The most common clinical signs were fever for 191 of cases. The splenomegaly and pallor were for 190 of cases (Table 2). The part of 67.3% of cases had a fever duration of ≤ 30 days (35) (Table 3). For the diagnostic delay and diagnosis test, 64.2% of cases were diagnosed in more than a month (122) and 204 of the cases were confirmed by serological examination. The treatment in 95.8% of cases was in a general mode (203) and the use of *Glunantime®* was in all cases (Table 3).

**Table 3:** clinical and entourage characteristics of VL cases in TTA region; 2009-2018

Variable (n)	Frequency	Percent (%)
**Fever duration (n=52)**		
≤30 days	35	67.3
>30 days	17	32.7
**Diagnostic test(n=264)**		
Skin smear	4	1.3
Serology test	204	67.1
Bone marrow smear	54	17.9
Biopsy	3	1.0
CBC	8	2.7
Delay of diagnosis*(n=190)		
≤1month	68	35.8
>1month	122	64.2
**Treatment (n=275)**		
Glunantime®	275	100.0
**Treatment modality (n=212)**		
Local	9	4.2
General	203	95.8
**Known cases in home (n=123)**		
No	116	94.3
Yes	7	5.7
**Known cases in surrounding (n=103)**		
No	101	98.1
Yes	2	1.9
**Suspects cases in home (n=102)**		
No	100	98.0
Yes	2	2.0
**Suspects cases in entourage (n=103)**		
No	98	95.1
Yes	5	4.9
**Dogs at home (n=112)**		
No	36	32.1
Yes	76	67.9
**Number of dogs (n=76)**		
>2dogs	11	14.5
≤ 2dogs	65	85.5
**Dogs in locality (n=106)**		
No	2	1.9
Yes	104	98.1
**Rodents in homes or entourage (n=79)**		
No	26	32.9
Yes	53	67.1

* The delay between the onset of symptoms and the confirmed diagnosis of VL disease

**Entourage characteristics:** the investigation of cases showed that 94.3% of 123 and 98% of 102 cases had no known or suspects cases around or in their homes. Also, 98.1% and 95.1% of 103 cases had no known or suspects cases in their entourage (Table 3). Concerning the reservoir of infection, 67.9% of 112 cases had dogs in their homes, with a number ≤2 dogs for 85.5% of 76 cases. In addition, 98.1% of cases had dogs (109) in their localities of residence. Rodents existed in and around homes for 67.1% of cases (53) (Table 3).

## Discussion

The results of our study indicate that VL infection continues to represent a significant public health concern in the TTA region from 2009 to 2018, particularly in Chefchaouen province. This situation has been previously documented in studies conducted between 1990 and 2014 and between 1997 and 2018 [12,17]. Indeed, the TTA region in addition to the Taza province (was included in the region before) in the north of Morocco, accounted for 65% of the national cases with 20% of the cases in Chefchaouen [12]. Chefchaouen province maintained the first endemic position not only within the TTA region, but also for the all Moroccan regions [12,17]. The provinces of Al-Hoceima and Ouezzane were reported endemic since 2000 as active national focuses of VL and continue to be according to our results [10]. In Morocco, geographically, VL disease follows two axes, from the north to the East and from the center to the south, which includes the endemic provinces in the TTA region [10]. The temporal distribution demonstrated that the incidence in TTA region was ranged from 1.8 case/100,000 inhabitants in 2010 to 0.4 case/100,000 inhabitants in 2018. In Algeria, an incidence below 0.5 case per 100,000 inhabitants was registered annually between 1998 and 2008 [20]. Furthermore, in Tunisia, the incidence was 1.04 cases/100,000 inhabitants, while in Turkey, the incidence ranged from 1.6 to 8.5 cases/100,000 inhabitants, which was higher than that observed in our country (Morocco) [21,22]. The variation in terms of incidence depends on the underreporting degree across the health system of countries [20]. For the number of cases, the maximum number was in the spring season and more specifically in March. In general, the monthly and seasonal distribution of cases is closely linked to climatic conditions [23]. Its influence the density of the vector, the host and the transmission cycle of the parasite [23]. In Morocco, the recovery of the vector's activity after the winter season and the increase in its activity in the summer affect the distribution of the sandfly vector infection [23].

This activity continues throughout the warm seasons [24]. The socio-economic profile was characterized by an age ≤ 5 years. Also, the male gender, rural origin and cases without profession were observed. The farmer was the most detected occupation for the cases with profession. In the first hand and for the variable of age, childhood was reported in Morocco and Maghreb studies [17,21,25]. The disease could be increased by malnutrition and low immune status [26,27]. It depends on the implication degree of *SLC11A1/NRAMP1* gene in the susceptibility or the resistance to VL infection among children [28]. Added to that, the VL disease creates a failure of the lipoprotein metabolism system [29]. Conversely, in France a study of VL program from 2001 to 2003 showed a mean age of 35 years of VL patient, however, 56% of them were adults with immunosuppression status [30]. On the other hand, male cases have been described in Morocco as well as in Sudan and Ethiopia [17,27,31]. This has been explained by physiological differences between the sexes [31]. In Brazil, the females had a double risk to have the VL infection due to *L. chagasi* [32]. For rural residence, it is known as an area of infection in Morocco [12,17]. The deterioration of living conditions supports the appearance of VL infection in this area [33]. Concerning the profession of cases, the patients without occupation are characterized by also low income, promoting the VL infection [34]. In the last and for the observed farmers patient in our study, the contact activities with reservoir animals and vectors increase their risk to be infected by the VL disease [31]. Demographically, the VL cases were characterized by a high number of inhabitants per household living. This may lead to the deterioration of living and housing conditions that contribute to the appearance of VL infection [33]. Also, the cases had lived in their localities for more than one year and hadn´t a secondary residence outside. The cases weren't displaced when they were affected. In Europe as well, the VL cases were reported without displacement or external travel [35].

Clinically, the cases initially presented with splenomegaly and pallor. The duration of the fever was ≤ 30 days. They were diagnosed in more than one month by serological testing and treated with the Glunantime® treatment. The VL parasite attacks first-line immune cells, including macrophages, which are present in high numbers in the spleen, causing splenomegaly [36]. In addition, the parasitism of the bone marrow leads to pancytopenia (a decrease in the production of blood elements) and the appearance of pallor [36]. The diagnosis test and treatment are supported by the Leishmaniasis health program [9]. In TTA region, the diagnosed delay for 64.2% was more than a month. In Algeria, this delay was 0-2 months for 50% cases and 30.1% of cases have exceeded 3 months [20]. This similar delay was observed in Tunisia and in Italy [21,37]. The lag time between signs and diagnosis may be referred to the misperception about the infection (for parents and doctors) [38] and access problems to health services [34]. Our study described the absence of suspected and known cases of VL in the home and entourage of the cases. This description was confirmed against the autochthonous origin of the infection [9]. Also, the Investigation showed the existence of dogs and rodents (VL reservoirs) in the entourage of cases. The domestic dog is the main reservoir of the *L. infantun* parasite in Morocco [39,40]. The dog is a source of blood food for the vector even in minimal parasitic charge and in the expectant form of the infection [41-43]. In other contexts, the presence of other reservoirs has been demonstrated, as in Brazil (cats) and Bangladesh (domestic cattle and goats) [44,45]. The existence of animals around and near houses promotes contamination by infection [46]. It is therefore necessary to update mammalogical and entomological surveys in northern Morocco to identify new reservoirs and possible vector species.

## Conclusion

In conclusion, our investigation was able to highlight the epidemiological profile of VL infection in the TTA region, with the persistence of Chefchaouen as the endemic province. Thus, the implementation of control and prevention measures against VL is necessary. Firstly, it is pertinent to monitor the entomological, mammalogical evolution of the vector and the reservoir in the region and throughout the country. At the provincial level, it will be important to activate the Integrated Vector Control Management Committee. Also, it is necessary to inform the community as well as the professionals of health about the VL control measures. Finally, it´s important to strengthen the research of the vaccine for humans, which has shown promising results which can be effective in the elimination of VL on national and international levels.

### What is known about this topic

- *The World Health Organization and the Morocco Ministry of Health and Social protection have insisted on in the adaptation of the preventing actions of the health program and the elimination of VL infection due to L. infantum the infection by 2030;*
- *The North of Morocco is still an endemic area for visceral leishmaniasis infection from the beginning of the prevention program in Morocco*.

### What this study adds

- *The visceral leishmaniasis infection continues to be an important health problem in North Morocco especially in Chefchaouen as a national and regional endemic area;*
- *The less of socio-economic cases level, the deterioration of living conditions and the presence of dogs and rodents (reservoir of VL) in the entourage house of cases, supports the appearance of visceral leishmaniasis infection;*
- *Our output will be used to adapt the preventive actions health program and eliminate the infection by 2030*.

## References

[1] World Health Organization (2020). Ending the neglect to attain the Sustainable Development Goals: a road map for neglected tropical diseases 2021-2030. WHO.

[2] World Health Organization Leishmaniasis.

[3] Pal M, Gutama KP, Steinmetz CH, Dave P (2022). Leishmaniasis: an emerging and re-emerging disease of global public health concern. AmJ Infect. Dis.

[4] Volpedo G, Huston RH, Holcomb EA, Pacheco-Fernandez T, Gannavaram S, Bhattacharya (2021). From infection to vaccination: reviewing the global burden, history of vaccine development, and recurring challenges in global leishmaniasis protection. Expert Rev Vaccines.

[5] Faucher B, Piarroux R (2011). Actualités sur les leishmanioses viscérales. Rev Med Interne.

[6] Bouchaud O, Consigny PH, Cot M, Le Loup G, Odermatt-Biays S (2019). Diseases Fiches. Méd des Voyages et Tropicale.

[7] World Health Organization (2010). Control of the leishmaniases. World Health Organization technical report series.

[8] World Health Organization EMRO (2023). Leishmaniasis. WHO.

[9] Ministry of health and social protection Guide to leishmaniasis control and prevention activities 2010. Morocco.

[10] Kahime K, Boussaa S, Nhammi H, Boumezzough A (2017). Urbanization of human visceral leishmaniasis in Morocco. Parasite Epidemiol Control.

[11] Kahime K, Bounoua L, Sereno D, El Hidan M, Messouli M (2020). Emerging and Re-Emerging Leishmaniases in the Mediterranean Area: What Can Be Learned from a Retrospective Review Analysis of the Situation in Morocco during 1990 to 2010?. Microorganisms.

[12] Mniouil M, Fellah H, Amarir Fa, Et-Touys A, Bekhti K, Adlaoui E (2017). Epidemiological characteristics of visceral leishmaniasis in Morocco (1990-2014). Acta Trop.

[13] El Hamouchi A, Ejghal R, Hida M, Lemrani M (2017). Intraspecific genetic variability in a population of Moroccan Leishmania infantum revealed by PCR-RFLP of kDNA minicircles. Acta Trop.

[14] El Miri H, Faraj C, Himmi O, Hmamouch A, Maniar S, Laaroussi T (2016). Cutaneous leishmaniasis in Ouazzane and Sidi Kacem provinces, Morocco (1997-2012). Bull Soc Pathol Exot.

[15] Ministry of health and social protection Bulletin of Epidemiology and Public Health.

[16] Ministry of health and social protection (2021). Department of Epidemiology and Disease Control. Bulletin of Epidemiology and Public Health.

[17] Hakkour M, El Alem MM, Hmamouch A, Rhalem A, Delouane B, Habbari K (2019). Leishmaniasis in Northern Morocco: Predominance of Leishmania infantum Compared to Leishmania tropica. Biomed Res Int.

[18] Barrett MP, Croft SL (2012). Management of trypanosomiasis and leishmaniasis. Br Med Bull.

[19] High Planning Commission Monograph of the Tangier Tetouan region Al-Hoceima.

[20] Adel A, Boughoufalah A, Saegerman C, Deken RD, Bouchene Z, Soukehal A (2014). Epidemiology of Visceral Leishmaniasis in Algeria: An Update. PLoS One.

[21] Aoun K, Jeddi F, Amri F, Ghrab J, Bouratbine A (2009). Current epidemiological data on visceral leishmaniasis in Tunisia. Med Mal Infect.

[22] Dujardin JC, Campino L, Cañavate C, Dedet J, Gradoni L (2008). Spread of vector-borne Diseases and Neglect of Leishmaniasis, Europe. Emerg Infect Dis.

[23] K Boussaa S, Ouanaimi F, Boumezzough A (2015). Species composition of phlebotomine sand fly fauna in an area with sporadic cases of Leishmania infantum human visceral leishmaniasis Morocco. Acta Trop.

[24] Coleman RE, Burkett DA, Sherwood V, Caci J, Spradling S, Jennings BT (2007). Impact of phlebotomine sand flies on U.S. Military operations at Tallil Air Base, Iraq: 2. Temporal and geographic distribution of sand flies. J Med Entomol.

[25] Amro A, Hamdi S, Lemrani M, Mouna I, Mohammed H, Mostafa S (2013). Moroccan Leishmania infantum: genetic diversity and population structure as revealed by multi-locus microsatellite typing. PLoS One.

[26] Mouttaki T, Maksouri H, El Mabrouki J, Merino-Espinosa G, Fellah H, Itri M (2018). Concomitant visceral and localized cutaneous leishmaniasis in two Moroccan infants. Infect Dis Poverty.

[27] Nackers F, Mueller YK, Salih N, Elhag MS, Elbadawi ME, Hammam O (2015). Determinants of Visceral Leishmaniasis: A Case-Control Study in Gedaref State, Sudan. PLoS Negl Trop Dis.

[28] Ejghal R, Hida M, Idrissi ML, Hessni AE, Lemrani M (2014). SLC11A1 polymorphisms and susceptibility to visceral leishmaniasis in Moroccan patients. Acta Trop.

[29] Bekaert ED, Dole E, Dubois DY, Bouma ME, Lontie JF, Kallel R (1992). Alterations in lipoprotein density classes in infantile visceral Leishmaniasis: presence of apolipoprotein SAA. Eur J Clin Invest.

[30] Basset D, Pratlong F, Rave C, Dereure J, Dedet J Leishmaniases in France: summary of data collected from 2001 to 2003 at the National Reference Center for Leishmania.

[31] Kirstein OD, Skrip L, Abassi I, Iungman T, Horwitz BZ, Gebresilassie A (2018). A fine scale eco-epidemiological study on endemic visceral leishmaniasis in north Ethiopian villages. Acta Trop.

[32] Evans T, Teixeira M, Mcauliffe I, Vasconcelos I, Vasconcelos A, Sousa A (1992). Epidemiology of Visceral Leishmaniasis in Northeast Brazil. J Infect Dis.

[33] Reis LL dos, Balieiro AA da S, Fonseca FR, Gonçalves MJF (2017). Changes in the epidemiology of visceral leishmaniasis in Brazil from 2001 to 2014. Rev Soc Bras Med Trop.

[34] Bastos Rolim Nunes BE, Leal TC, Silva de Paiva JP, da Silva LF, do Carmo RF, Machado MF (2019). Social determinants of mortality due to visceral leishmaniasis in Brazil (2001-2015): an ecological study. Rev Soc Bras Med Trop.

[35] Ehehalt U, Schunk M, Jensenius M, van Genderen PJJ, Gkrania-Klotsas E, Chappuis F (2014). Leishmaniasis acquired by travellers to endemic regions in Europe: A EuroTravNet multi-centre study. Travel Med Infect Dis.

[36] McGwire BS, Satoskar AR (2014). Leishmaniasis: clinical syndromes and treatment. QJM.

[37] Pagliano P, Rossi M, Rescigno C, Altieri S, Coppola MG, Gramiccia M (2003). Mediterranean visceral leishmaniasis in HIV-negative adults: a retrospective analysis of 64 consecutive cases (1995-2001). J Antimicrob Chemother.

[38] Bouratbine A, Moussa H, Aoun K, Ben Ismail R (1998). Anthropologic research and understanding pediatric visceral leishmaniasis in Tunisia. Bull Soc Pathol Exot.

[39] Rhajaoui M (2011). Human leishmaniasis in Morocco: a nosogeographical diversity. Pathol Biol (Paris).

[40] Kahime K, Boussaa S, Bounoua L, Fouad O, Messouli M, Boumezzough A (2014). Leishmaniasis in Morocco: diseases and vectors. Asian Pac J Trop Dis.

[41] Rab MA, Frame IA, Evans DA (1995). The role of dogs in the epidemiology of human visceral leishmaniasis in northern Pakistan. Trans R Soc Trop Med Hyg.

[42] Pinelli E, Killick-Kendrick R, Wagenaar J, Bernadina W, Real G (1994). Ruitenberg J. Cellular and humoral immune responses in dogs experimentally and naturally infected with Leishmania infantum. Infect Immun.

[43] Cabral M, O´grady J, Alexander J (1992). Demonstration of Leishmania specific cell mediated and humoral immunity in asymptomatic dogs. Parasite Immunol.

[44] Metzdorf IP, da Costa Lima MS, de Fatima Cepa Matos M, de Souza Filho AF, de Souza Tsujisaki RA, Franco KG (2017). Molecular characterization of Leishmania infantum in domestic cats in a region of Brazil endemic for human and canine visceral leishmaniasis. Acta Trop.

[45] Alam MZ, Rahman MM, Akter S, Talukder MH, Dey AR (2018). An investigation about the possible role of cattle and goats as reservoir hosts for Leishmania donovani in Bangladesh. J Vector Borne Dis.

[46] Singh SP, Hasker E, Picado A, Gidwani K, Malaviya P, Singh RP (2010). Risk factors for visceral leishmaniasis in India: further evidence on the role of domestic animals. Trop Med Int Health.

